# Diseases of the Eye

**Published:** 1893-06

**Authors:** 


*Diseases of the Eye.* By G. E. DE SCHWEINITZ, M.D. Pp. 641. Philadelphia: W. B. SAUNDERS. 1892.

This addition to the large number of text-books already published for instruction on diseases of the eye presents in the method of its compilation no features that require especial notice. It is simply and clearly written; without going very fully into descriptive or theoretical detail, it contains all that the student is likely to require. The various diseases of the eye are well described, together with all their important subdivisions. Treatment receives its full share of attention; for this reason the book can be recommended to the family practitioner. Thus, ophthalmia in its various and serious forms is well dealt with, and the treatment recommended in trachoma includes most of our recent advances on this subject. The numerous anomalies in the movements of the eyeballs are carefully explained, and diseases at the fundus of the eyeball receive sufficient attention.

These are mentioned, not as exceptional chapters, but as types of the standard of the whole book, which, as its author has intended, is adapted rather for students and practitioners who desire to begin the study of ophthalmology than for those who, with a good knowledge of the subject, require a book for reference or for physiological or mathematical descriptions of detail.

